# Altered Cerebellar White Matter Integrity in Patients with Mild Traumatic Brain Injury in the Acute Stage

**DOI:** 10.1371/journal.pone.0151489

**Published:** 2016-03-11

**Authors:** Zhongqiu Wang, Wenzhong Wu, Yongkang Liu, Tianyao Wang, Xiao Chen, Jianhua Zhang, Guoxing Zhou, Rong Chen

**Affiliations:** 1 Department of Radiology, Affiliated Hospital of Nanjing University of Chinese Medicine, 155 Hanzhong Road, Nanjing 210029, Jiangsu, China; 2 Department of Acupuncture & Rehabilitation, Affiliated Hospital of Nanjing University of Chinese Medicine, 155 Hanzhong Road, Nanjing 210029, Jiangsu, China; 3 Department of Radiology, The Fifth People's Hospital of Shanghai, Fudan University, Shanghai 200240, China; 4 Department of Radiology, Shanghai East Hospital, Tongji University School of Medicine, Shanghai 200120, China; 5 Department of Diagnostic Radiology and Nuclear Medicine, University of Maryland School of Medicine, Baltimore, MD 21201, United States of America; University of North Carolina, UNITED STATES

## Abstract

**Background and Purpose:**

Imaging studies of traumatic brain injury demonstrate that the cerebellum is often affected. We aim to examine fractional anisotropy alteration in acute-phase mild traumatic brain injury patients in cerebellum-related white matter tracts.

**Materials and Methods:**

This prospective study included 47 mild traumatic brain injury patients in the acute stage and 37 controls. MR imaging and neurocognitive tests were performed in patients within 7 days of injury. White matter integrity was examined by using diffusion tensor imaging. We used three approaches, tract-based spatial statistics, graphical-model-based multivariate analysis, and region-of-interest analysis, to detect altered cerebellar white matter integrity in mild traumatic brain injury patients.

**Results:**

Results from three analysis methods were in accordance with each other, and suggested fractional anisotropy in the middle cerebellar peduncle and the pontine crossing tract was changed in the acute-phase mild traumatic brain injury patients, relative to controls (adjusted p-value < 0.05). Higher fractional anisotropy in the middle cerebellar peduncle was associated with worse performance in the fluid cognition composite (r = -0.289, p-value = 0.037).

**Conclusion:**

Altered cerebellar fractional anisotropy in acute-phase mild traumatic brain injury patients is localized in specific regions and statistically associated with cognitive deficits detectable on neurocognitive testing.

## Introduction

Traumatic Brain Injury (TBI) affects more than 1.5 million individuals annually in the United States, and 10 million individuals annually worldwide [1]. The long-term disabilities associated with TBI include cognitive, psychological, motor, and sensory deficits. Among TBI patients, about 80% of them are classified as mild TBI (mTBI), 10% are moderate, and 10% are severe [2]. Approximately 15% of patients with mTBI have persistent neurological symptoms [3]. The burden of mortality and morbidity associated with TBI makes it a pressing public health and medical problem.

Imaging studies of TBI demonstrate that cerebellum is often affected even when the initial injury does not directly involve this region [4–6]. A subset of TBI patients could develop delayed-onset cerebellar syndromes with three weeks to two years after injury [7]. Studies of delayed-onset cerebellar syndromes reported that these patients had lesions in thalamus or brainstem [7, 8]. This suggests that TBI-induced syndrome could be associated with the pathway involving the cerebellum. Therefore, it is of great importance to understand damage involving cerebellum-related white matter tracts in TBI patients.

White matter integrity can be examined by using diffusion tensor imaging (DTI). DTI uses magnetic resonance imaging (MRI) in different diffusion-sensitizing gradient directions, and is capable of detecting microstructural white matter changes. DTI has been used to characterize brain injuries in mTBI [3, 9–12]. Fractional anisotropy (FA) is a widely used DTI-derived measure that describes the degree of directionality of diffusion. Using FA as a marker of traumatic axonal injury has been directly validated by comparing FA to immunohistochemical indicators of axonal injury in an animal model of traumatic brain injury [13]. Using analysis methods such as region-of-interest (ROI) analysis and tract-based spatial statistics (TBSS), researchers have shown many white matter tracts are damaged in mTBI and the microstructural integrity of these tracts correlates with behavioral and neurocognitive measures [3, 9–12, 14]. However, DTI studies rarely examined cerebellum-related white matter tracts for acute-stage mTBI patients in the general population.

We hypothesize that FA alteration would be present in acute-phase mTBI patients in cerebellum-related white matter tracts. We will use three approaches, TBSS, graphical-model-based multivariate analysis (GAMMA), and ROI analysis, to test this hypothesis. TBSS [15] is a powerful method to analyze DTI measures. TBSS is a voxelwise, mass univariate, general linear model based method to detect voxels characterizing group differences. Tract-based GAMMA [16, 17] and ROI analysis are complementary to TBSS; and provide further information about microstructural white matter changes. First, GAMMA is a Bayesian multivariate approach complementary to the mass-univariate general linear model based methods. Relative to TBSS, GAMMA can identify voxels which are predictive of the group-membership variable (mTBI or controls) at the individual level, instead of at the group level. Second, FA changes could be distributed across regions. Combining tract space analysis and ROI analysis has the potential to detect subtle changes in FA and facilitate future meta-analyses [18].

## Methods

### Participants

**Fig 1:**
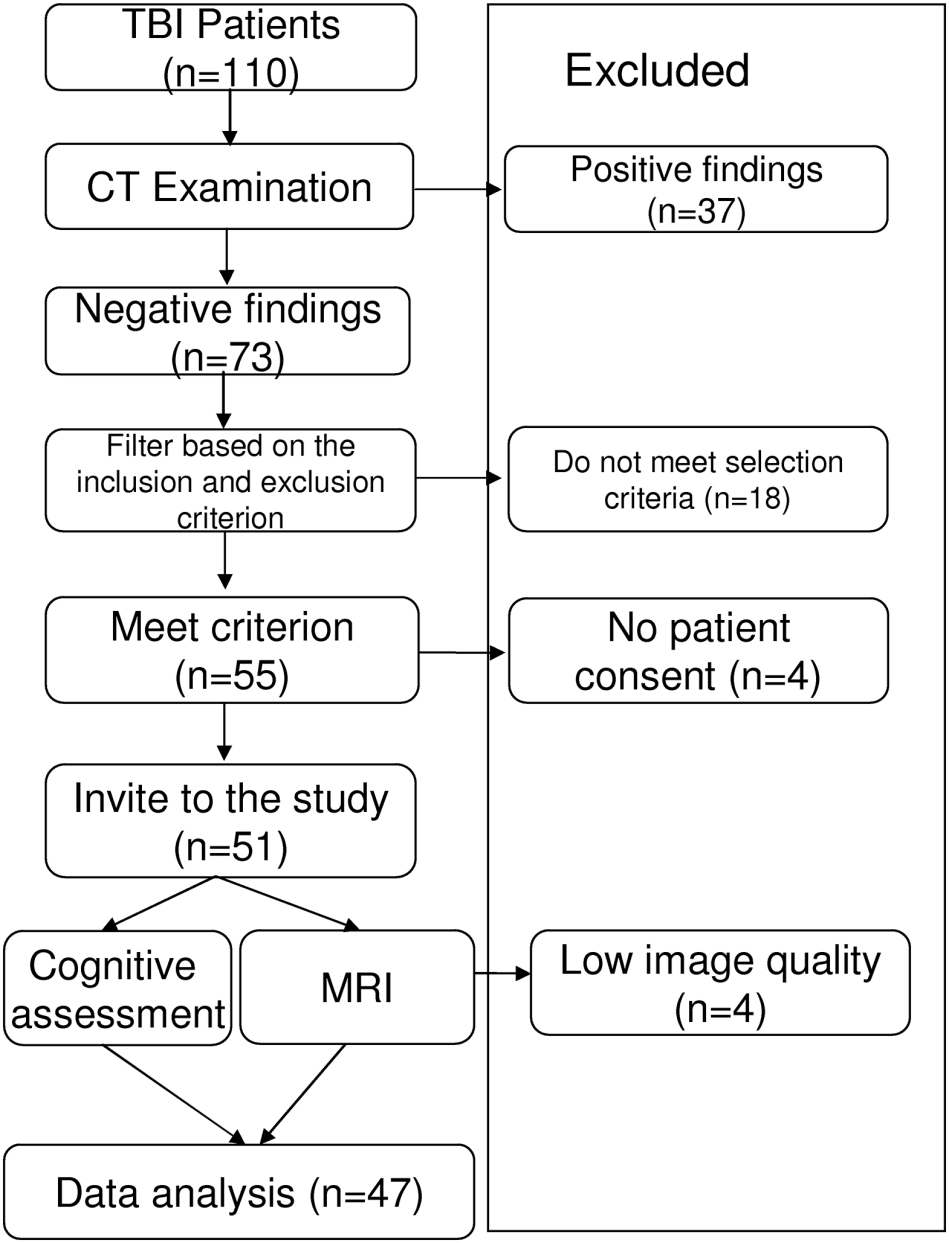
A diagram of the recruitment workflow.

Local research ethics committee approved this study. All of participants provided written informed consent. This two-year prospective study included 47 mTBI patients and 37 normal controls. The recruitment workflow is depicted in Fig 1.

The diagnosis of mTBI was established by using the criteria of the American Congress of Rehabilitative Medicine (ACRM) for mild brain injury [19]. In the ACRM definition of mTBI, a subject is considered to have mTBI if any one of the following symptoms following external application of force to the brain: 1) any period of loss of consciousness, 2) any loss of memory for events immediately before or after the accident, 3) any alteration in mental state at the time of the accident, or 4) focal neurologic deficit(s) that may or may not be transient. The ACRM definition of mTBI includes injuries in which loss of consciousness is 30 minutes or less, the Glasgow Coma Scale (GCS) score at 30 minutes after injury is 13–15, and the duration of post-traumatic amnesia is no longer than 24 hours.

The exclusion criteria were: 1) history of significant ear surgery, 2) penetrating head injury, 3) pregnancy, 4) history of dementia or mental disorder, 5) uremia, liver cirrhosis, heart failure, pulmonary edema, coagulopathy and renal dysfunction, 6) ischemic and hemorrhagic stroke, 7) in vivo magnetic implants (such as iron, or cochlear implants, vascular clips, etc.) or pacemaker, 8) the patient either died or had received cardiopulmonary resuscitation before arrival at the hospital, and 9) positive CT findings.

MR imaging and neurocognitive tests were performed in mTBI patients within 7 days of injury. This study included mTBI patients in the acute stage. The control group included age- and gender-matched healthy subjects with no history of neurological or psychiatric illness, and no prior TBI. All participants (mTBI and controls) were right-handed.

### MRI protocol

MR data were acquired with a Philips Achieva 3.0T TX MRI scanner (Royal Philips, Amsterdam, Netherlands). The MR protocol included anatomical imaging (T1 and T2) and diffusion tensor imaging. High-resolution T1-weighted structural images were acquired with a MPRAGE sequence: TR/TE = 8.2 ms/3.5 ms; flip angle = 8 degree; slice thickness = 1mm, voxel size = 1 mm *1 mm, FOV = 256*256. Diffusion data were acquired with a single-shot echo-planar sequence (TR/TE = 9,000 ms/90 ms, slice thickness = 2mm, in-plane image resolution = 2 mm *2 mm, field of view = 256 * 256 mm). 32 non-collinear diffusion gradient directions were used with b = 1,000 s/mm^2^, and one b = 0 s/mm^2^ reference image. These imaging parameters were consistent with the common data element standard for TBI [20].

### Neuropsychological assessment

Neuropsychological tests were administrated within 24 hours of MR imaging. The neuropsychological tests included the flanker inhibitory control and attention test (Flanker), the list sorting working memory test (List Sorting), the dimensional change card sort test (DCCS), pattern comparison processing speed (Pattern Comparison), the picture sequence memory test (PSMT) [21]. The Flanker task measures executive function, specifically inhibitory control and attention. List Sorting measures working memory. The DCCS measures executive function, specifically tapping cognitive flexibility. The Pattern Comparison test is a measure of speed of processing. The PSMT is a measure of episodic memory. The NIH Toolbox fluid cognition composite score is generated based on Flanker, DCCS, PSMT, List Sorting, and Pattern Comparison. It measures the capacity for new learning and information processing in novel situations. We used the NIH Toolbox fluid cognition composite score as the primary neuropsychological variable.

### Voxel-based analysis of FA

DTI images were preprocessed by using FMRIB Software Library (FSL) [22]. For motion and eddy current correction, diffusion-weighted images were registered to the b0 image using an affine registration algorithm. Brain Extraction Tool [22] was used to exclude non-brain tissues in the T1- and diffusion-weighted data. We visually inspected the skull-stripped images. If necessary, we manually corrected the skull-stripping error. FA images were generated using the Diffusion Toolbox [22].

FA images were analyzed using TBSS. The TBSS procedure was as follows. First, FA images were normalized to the standard FMRIB58 FA template using the nonlinear registration algorithm in FSL [22]. Then we averaged the normalized FA images to create the mean FA map. The mean FA map was the input to the tract skeleton generation. The skeleton of a tract is a single line (or surface) running down the center of this tract. For the FA skeleton generation, a FA threshold of 0.2 was used to exclude voxels which are primarily gray-matter or cerebrospinal fluid. Then we projected individual subject’s FA onto the FA skeleton. We use permutation methods in FSL (the FSL randomise procedure) to test FA differences between the mTBI and control groups. The WM integrity differences were investigated by using the threshold-free cluster enhancement at p-value < 0.05 (5000 permutations) fully corrected for multiple comparisons. Since we were interested in the cerebellum-related white matter tracts, a cerebellum and brain stem mask in the MNI space was used to restrict our analysis.

### Graphical-model-based multivariate analysis of FA

Graphical-model-based multivariate analysis (GAMMA) [16, 17] is a voxel-based Bayesian multivariate approach for biomarker detection. GAMMA is complementary to the mass-univariate general linear model based approaches. Relative to the mass-univariate general linear model based approaches, GAMMA is a multivariate analysis method and can detect combinational interactions among brain regions and the group-membership variable.

The input to GAMMA is the skeletonized FA maps and the group-membership variable (mTBI or controls). Outputs of GAMMA are a set of brain regions which are jointly predictive of the group-membership variable. Each brain region is a biomarker. GAMMA uses Bayesian regularization to address the multiple-comparison problem. We used the GAMMA algorithm implemented in [23] for GAMMA analysis.

### ROI-based analysis of FA

All input to and output from the cerebellum is by way of the three cerebellar peduncle (superior, middle, and inferior) [24]. Therefore, our ROIs were the middle cerebellar peduncle, the pontine crossing tract (a part of middle cerebellar peduncle), the left and right superior cerebellar peduncle, and the left and right inferior cerebellar peduncle. ROIs were defined using the Johns Hopkins University white matter atlas [25]. Our ROI analysis was based on the skeletonized FA map and the Johns Hopkins University white matter atlas. Both were in the same stereotaxic space: the Montreal Neurological Institute (MNI) space. There were total six ROIs in our analysis. For each ROI, we calculated the average FA value in the skeletonized FA map. Then we used independent 2-group Mann-Whitney U test (function wilcox.test in R) to identify ROIs whose FA values were different between the mTBI and controls groups. The false discovery rate correction was used to correct for multiple testing.

## Results

### Participant characteristics

For the mTBI group, the mean age was 30.0 years (standard deviation (SD) 6.8, range 18–45). The female:male ratio was 15:32. For the control group, the mean age was 31.4 years (SD 8.7, range 21–49). The female:male ratio was 16:21. There were no significant differences in age (p-value = 0.319 based on the two-sample *t*-test) or female:male ratio (p-value = 0.203 based on the chi-square test).

For mTBI patients, the cause of injury included traffic accidents (26%), falls (9%), sports-related accidents (6%), and objects striking the head (60%). The injury severity in mTBI patients was mild: 96% with GCS = 15.

### Structural imaging data

T1- and T2-weighted images were reviewed by an experienced neuroradiologist (ZQW) in order to identify structural abnormalities, including assessment for evidence of hemorrhage. Reviewers were blinded to the group membership and clinical information. These were found to be free of T1- and T2-MRI abnormalities for both mTBI and controls.

### Voxel-based analysis of FA

**Fig 2:**
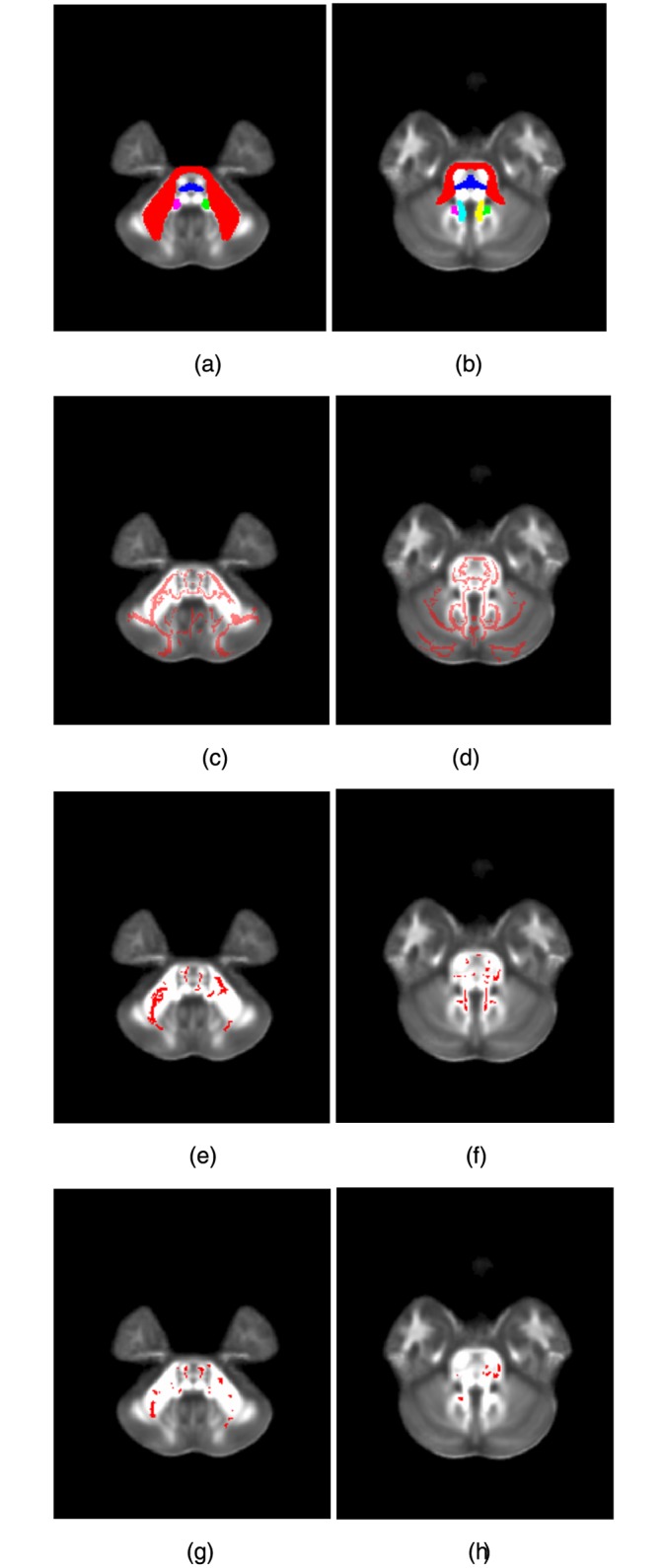
Regions where the mTBI group showed significant higher FA compared to controls. (a) and (b): The six ROIs defined in the Johns Hopkins University white matter atlas, superimposed on the MNI FA template. Red is the middle cerebellar peduncle; blue is the pontine crossing tract; green is the right inferior cerebellar peduncle; purple is the left inferior cerebellar peduncle; yellow is the right superior cerebellar peduncle; and cyan is the left superior cerebellar peduncle. For (a) and (b), the slice numbers are 36 and 45, respectively. (c) and (d): The FA skeleton (slice number = 36 and 45). (e) and (f): The voxels characterizing mTBI detected by TBSS, superimposed on the MNI FA template (slice number = 36 and 45). (g) and (h): The voxels characterizing mTBI detected by GAMMA, superimposed on the MNI FA template (slice number = 36 and 45).

The TBSS results are shown in Fig 2. The FA skeleton generated by TBSS is in Fig 2c and 2d. The slice numbers for Fig 2c and 2d are 36 and 45, respectively. Fig 2a and 2b are the middle cerebellar peduncle, the pontine crossing tract, the right inferior cerebellar peduncle, the left inferior cerebellar peduncle, the right superior cerebellar peduncle, and the left superior cerebellar peduncle, defined in the Johns Hopkins University white matter atlas, superimposed on the MNI FA template. In Fig 2e and 2f, regions of higher FA in the mTBI group revealed by TBSS (corrected p-value < 0.05) are shown in red, superimposed on the MNI FA template. Elevated FA was particularly concentrated in the middle cerebellar peduncle. We did not observe reduced FA in the mTBI group relative to controls. There were 1972 voxels which demonstrated significantly higher FA in the mTBI group. 88% of these voxels were in the six ROIs. Among them, 60% were in the middle cerebellar peduncle, 8% were in the pontine crossing tract, 6% were in the right inferior cerebellar peduncle, 5% were in the left inferior cerebellar peduncle, 7% were in the right superior cerebellar peduncle, and 4% were in the left superior cerebellar peduncle.

### GAMMA-based analysis of FA

The GAMMA-based analysis results are shown in Fig 2g and 2h. In Fig 2g and 2h, regions characterizing mTBI are shown in red, superimposed on the MNI FA template. These voxels were mainly in the middle cerebellar peduncle. There were 1082 voxels characterizing mTBI. 83% of these voxels were in the six ROIs. Among them, 54% were in the middle cerebellar peduncle, 17% were in the pontine crossing tract, 4% were in the right inferior cerebellar peduncle, 6% were in the left inferior cerebellar peduncle, 1% were in the right superior cerebellar peduncle, and 1% were in the left superior cerebellar peduncle.

### ROI-based analysis of FA

**Fig 3:**
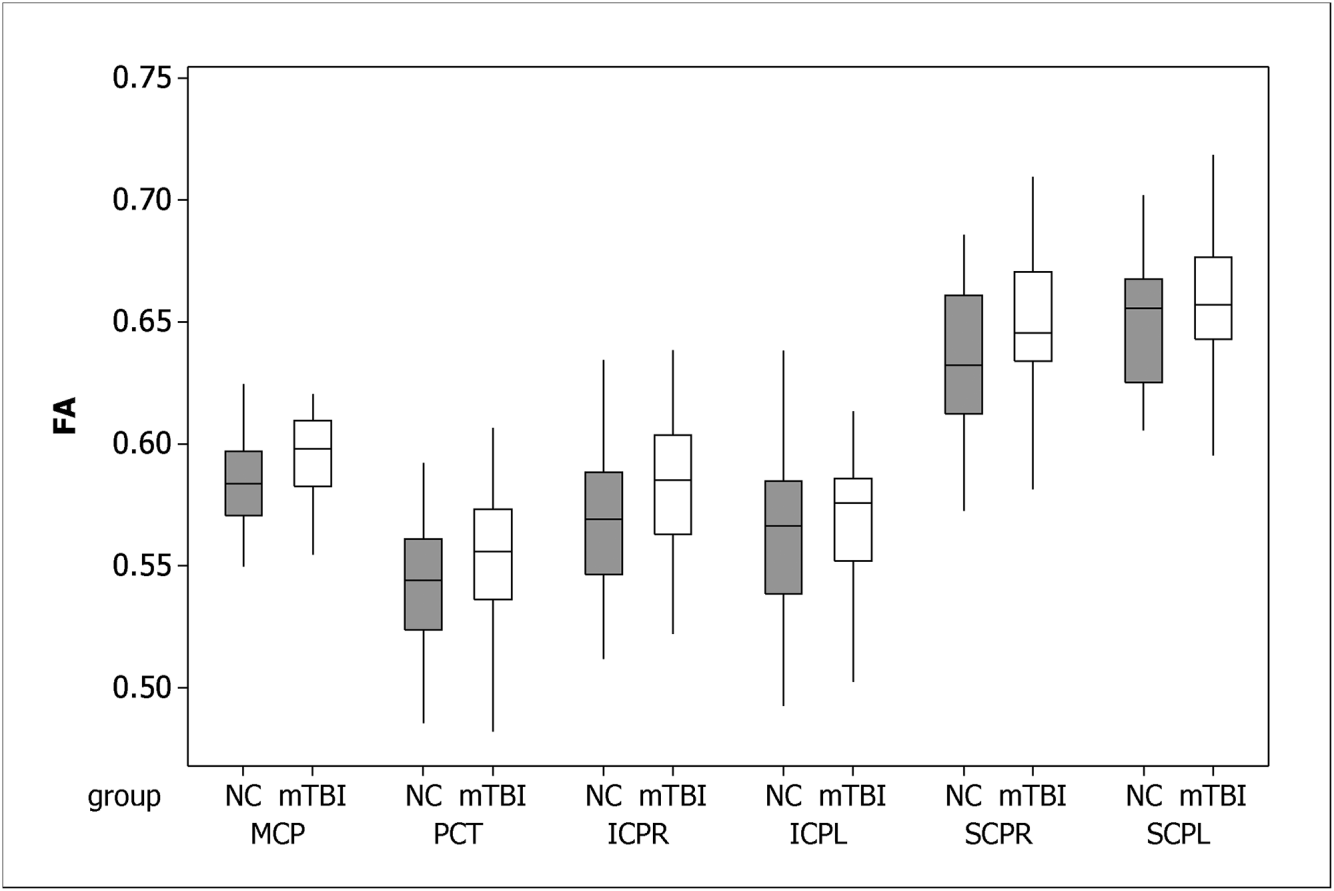
Boxplots of regional FAs. MCP: middle cerebellar peduncle; PCT: the pontine crossing tract; ICPR: inferior cerebellar peduncle, right; ICPL: inferior cerebellar peduncle, left; SCPR: superior cerebellar peduncle, right; SCPL: superior cerebellar peduncle, left.

The box plots of regional FAs are in Fig 3. The result of ROI-based analysis of FA is summarized in Table 1. We found that FA was higher in the mTBI group for all ROIs, relative to controls. FA was significantly higher (FDR adjusted p-value < 0.05) in the mTBI patients relative to controls in the middle cerebellar peduncle and the pontine crossing tract. For the middle cerebellar peduncle, the median and interquartile range (IQR) for controls and mTBI were 0.584(0.026) and 0.598(0.027), respectively. For the pontine crossing tract, the median and IQR for controls and mTBI were 0.544(0.037) and 0.556(0.037), respectively. The right inferior cerebellar peduncle and right superior cerebellar peduncle also showed higher FA. However, their FDR adjusted p-values were greater than 0.05. There were no significant differences between groups in the left inferior cerebellar peduncle and left superior cerebellar peduncle.

**Table 1:** The p-values of the ROI-based analysis of FA.

ROI	Controls (n = 37, median (IQR))	mTBI (n = 47, median (IQR))	Raw p-value	Adjusted p-value
middle cerebellar peduncle*	0.584(0.026)	0.598(0.027)	0.006	0.038
the pontine crossing tract*	0.544(0.037)	0.556(0.037)	0.016	0.049
inferior cerebellar peduncle, right	0.569(0.042)	0.585(0.041)	0.044	0.066
inferior cerebellar peduncle, left	0.566(0.046)	0.576(0.034)	0.099	0.119
superior cerebellar peduncle, right	0.632(0.048)	0.646(0.036)	0.043	0.066
superior cerebellar peduncle, left	0.655(0.042)	0.657(0.033)	0.242	0.242

Raw p-values are computed based on the independent 2-group Mann-Whitney U Test. Adjusted p-values are generated using FDR.

*: ROIs with adjusted p-value < 0.05.

### Correlation analysis

We performed the Spearman correlation analysis between ROI FA and neuropsychological assessment in patients with mTBI (function cor.test in R). We found that FA in the middle cerebellar peduncle was associated with the fluid cognition composite score, with Spearman correlation coefficient = -0.289, p-value = 0.037. This indicated worse performance in the fluid cognition composite was associated with higher FA in the middle cerebellar peduncle. FA in the pontine crossing tract was not associated with the result of the fluid cognition composite score.

### Effects of injury type

Our study included mTBI patients with different injury types. We conducted two analyses to examine the effect of injury type. First, we performed one-way ANOVA (function aov in R) to test whether there were significant differences in regional FAs across injury types. In this analysis, the outcome variable was a regional FA such as FA in the middle cerebellar peduncle; and groups were defined by the injury type. For the injury type, we coded ‘striking’ as 1, ‘traffic’ as 2, ‘falls’ as 3, and ‘sports’ as 4. The *p*-values based on one-way ANOVA were 0.697 (the middle cerebellar peduncle), 0.996 (the pontine crossing tract), 0.118 (the right inferior cerebellar peduncle), 0.892 (the left inferior cerebellar peduncle), 0.410 (the right superior cerebellar peduncle), 0.818 (the left superior cerebellar peduncle). No regions demonstrated significant differences in regional FAs across injury types.

Second, we used stepwise regression with forward selection to investigate the effect of injury type (function stepAIC in R). In stepwise regression, the dependent variable was a regional FA such as FA in the middle cerebellar peduncle. Let *C* represent whether a subject is an mTBI patient or control. The potential predictors were *C* and the injury type. For the injury type, we coded ‘no injury’ as 0, ‘striking’ as 1, ‘traffic’ as 2, ‘falls’ as 3, and ‘sports’ as 4. In stepwise regression with forward selection, we started with no variables in the model, then added a variable if it improved the model fitness metric, and repeated this process until none improved the model. If dividing mTBI patients into sub-types (different injury types) is more predictive of regional FA than *C*, stepwise regression will generate a model that includes the injury type as the final predictor, instead of *C*. We found that for all six ROIs, the final models only included *C* as the predictor. We observed that dividing mTBI patients into sub-types (different injury types) was not more predictive of regional FA than *C*.

## Discussion

This study aimed to detect altered cerebellar FA in the acute-phase mTBI patients. Three FA analysis methods (TBSS, tract-based GAMMA, and ROI-based analysis) were used to examine cerebellar peduncles. These methods provided complementary information about white matter changes. A consistent pattern revealed by all three methods suggested FA in the middle cerebellar peduncle and the pontine crossing tract was changed in the acute-phase mTBI patients, relative to controls. Higher FA in the middle cerebellar peduncle was associated with worse performance in the fluid cognition composite.

Accumulating evidence indicates the cerebellum is affected in TBI patients. Many imaging studies of TBI address the effect of TBI on the cerebellum. Cerebellar atrophy following TBI is reported in [6, 26]. Gale et al. [26] used voxelwise brain morphometry to evaluate changes in grey matter concentration following TBI (ranging from mild to severe, in the chronic phase). They reported TBI patients had decreased grey matter concentration in the cerebellum, relative to controls. In a PET study of 58 patients with brain injury (44 traumatic, 8 anoxic, 4 vascular and 2 toxic injuries) and age- and sex-matched controls [27], Lupi et al. reported a hypermetabolic cerebellar vermis in the brain injury group, regardless of the nature of the trauma. In a perfusion imaging study of 27 mTBI patients in the chronic phase and age-matched controls [28], voxelwise analysis of relative cerebral blood flow maps demonstrated decreased perfusion in the cerebellum in the group with mild TBI relative to the controls. Our study found that altered cerebellar FA in mTBI patient in the acute stage. All these imaging studies suggest that cerebellum is affected in TBI patients.

Our study is related to other mTBI imaging studies involving the cerebellum. Mac Donald et al. [10] examined DTI data of 63 U.S. military personnel who experienced mTBI and primary blast exposure and a control group comprised of 21 military personnel who experienced blast exposure but did not have mTBI. They found that the mTBI group showed marked DTI abnormalities in the middle cerebellar peduncles. Their study centers on the military population as opposed to the civilian population that was investigated in our study. In a retrospective study, Alhilahi et al. [11] analyzed DTI data of 30 mTBI patients with vestibular symptoms and 25 mTBI patients with ocular convergence insufficiency. Control subjects consisted of 39 mTBI patients without vestibular abnormalities and 17 mTBI patients with normal ocular convergence. They found that relative to controls, mTBI patients with vestibular symptoms had decreased FA values in the cerebellum and fusiform gyri. Alhilahi’s study didn’t compare acute-phase mTBI patients with normal controls. Wang et al. [12] analyzed DTI data of 28 patients with mild to severe traumatic axonal injury (23 of 28 had severe brain injury) and 20 age- and sex-matched healthy controls and found that 9 fiber tracts, including cerebral peduncular fibers, showed acute structural damage. Their study population consisted primarily of severe TBI subjects. In summary, although there are studies reporting altered cerebellar white matter integrity in mTBI patients, our study is unique in that it centers on cerebellar FA changes in acute-stage mTBI civilian patients and our study has a relatively large sample size.

Accumulating evidence indicates that the cerebellum is not only implicated in motor function, but also involved in cognitive function [29–31]. In this study, we found that in mTBI patients, higher FA in the middle cerebellar peduncle was associated with worse performance in the fluid cognition composite. The middle cerebellar peduncle is the primary pathway by which the cerebral cortex influences the cerebellum. The major sources of corticopontocerebellar fibers include the regions near the central sulcus, prefrontal cortices, and limbic cortices. This suggests that damage of the middle cerebellar peduncle could affect cognitive function. The fluid cognition composite score measures the capacity for new learning and information processing in novel situations. Our findings suggest that altered cerebellum FA in the acute-phase mTBI patients is associated with impaired capacity for new learning and information processing.

In this study, we found mTBI patients have higher FA in the middle cerebellar peduncle and the pontine crossing tract, relative to controls. This is consistent with other acute-phase mTBI studies [32–34]. One possible explanation of increased FA in the acute stage is axonal swelling. Axonal swelling could cause a net movement of water into the intracellular space; and restricts the interstitial space, causing an increase in anisotropic diffusion. This will lead to higher FA. A clear picture of FA change in the acute mTBI patients has not yet emerged from neuroimaging studies [9]. There are studies reporting acute mTBI patients have reduced FA. In a meta-analysis [9], Eierud et al. found that elevated anisotropy values are more frequently reported in acute mTBI studies, while reduced anisotropy values are reported more frequently in post-acute mTBI studies.

In this study, we used three different FA analysis methods to detect cerebellar FA changes. These analysis methods are complementary to each other. TBSS is one of the most widely used FA analysis methods. TBSS is based on mass-univariate general linear models. Tract-based GAMMA combines tract space analysis and GAMMA; and detects biomarkers which distinguish subjects at the individual level, instead of at the group level. Compared to voxel-based methods, ROI-based analysis has the potential to increase signal-to-noise ratio. All three methods identified similar brain regions of FA changes.

Despite the strength of this study, including the relatively large sample size and three different FA analysis methods to detect cerebellar FA changes, these data should be interpreted in light of potential limitations. One limitation is that our study is hypothesis-driven and centers on cerebellar FA changes. We found that higher FA in the middle cerebellar peduncle in mTBI patients was associated with worse performance in the fluid cognition composite. We cannot attribute variability in the fluid cognition composite to abnormalities in the middle cerebellar peduncle alone because we did not examine cerebral FA changes. This study can be expanded to investigate the association between cerebral and cerebellar FA changes. Another limitation is that our study is cross-sectional, and cannot examine temporal changes. We observed altered cerebellar FA in the acute-phase mTBI patients and higher FA in the middle cerebellar peduncle was associated with worse performance in the fluid cognition composite. An important question is whether this pattern still exists in sub-acute or chronic phase mTBI patients. We can address this problem using a longitudinal design. We plan to expand our work to investigate the cerebellar FA trajectories and their association with neurocognitive performance.

This study is based on DTI which is one of the most widely used methods to assess white matter integrity. Cerebellar white matter function can be assessed using other techniques such as perfusion imaging. One direction of our future work is to use multimodal imaging. Multimodal imaging combines different imaging techniques which provide complementary information about white matter integrity.

## Conclusions

This study is a comprehensive investigation of cerebellar FA abnormality in the acute-phase mTBI patients. We found that FA in the middle cerebellar peduncle and the pontine crossing tract was changed in the acute-phase mTBI patients, relative to normal controls. This finding is of great importance to understand damage involving cerebellum-related white matter tracts in TBI patients.

## Appendix

To examine the effect of injury type, we conducted an auxiliary analysis. Among the 47 mTBI patients in our original analysis, 4 of them had fall injuries and 3 of them had sports-related accident injuries. In the auxiliary analysis, these 7 subjects were excluded. The auxiliary analysis included 40 mTBI patients and 37 normal controls. In the mTBI group, the mean age was 29.4 years (SD 6.9). The female:male ratio was 14:26. In the control group, the mean age was 31.4 years (SD 8.7). The female:male ratio was 16:21. There were no significant differences in age (p-value = 0.26 based on the two-sample t-test) or female:male ratio (p-value = 0.46 based on the chi-square test).

In the auxiliary analysis, we performed ROI-based analysis of FA. We found that FA was higher in the mTBI group for all six ROIs, relative to normal controls. The raw p-values were 0.018 (middle cerebellar peduncle), 0.020 (the pontine crossing tract), 0.012 (inferior cerebellar peduncle, right), 0.092 (inferior cerebellar peduncle, left), 0.106 (superior cerebellar peduncle, right), and 0.380 (superior cerebellar peduncle, left). FA was significantly higher (FDR adjusted p-value < 0.05) in the mTBI patients relative to controls in three ROIs: the middle cerebellar peduncle, the pontine crossing tract, and the right inferior cerebellar peduncle. We found that FA in the middle cerebellar peduncle was associated with the fluid cognition composite score (Spearman correlation coefficient = -0.404, p-value = 0.023). FAs in the pontine crossing tract and the right inferior cerebellar peduncle were not associated with the fluid cognition composite score. In summary, we found that the result of auxiliary analysis is very similar to that in our original analysis. Excluding mTBI subjects with injury type as “falls” and “sports-related accidents” from our original analysis didn’t have a significant impact on the results.

## References

[1] Hyder AA, Wunderlich CA, Puvanachandra P, Gururaj G, Kobusingye OC. The impact of traumatic brain injuries: a global perspective. NeuroRehabilitation 2007;22(5):341–53. 18162698

[2] Kushner D. Mild traumatic brain injury: toward understanding manifestations and treatment. Arch Intern Med 1998;158(15):1617–24. 9701095 10.1001/archinte.158.15.1617

[3] Shenton ME, Hamoda HM, Schneiderman JS, Bouix S, Pasternak O, Rathi Y, et al A review of magnetic resonance imaging and diffusion tensor imaging findings in mild traumatic brain injury. Brain Imaging Behav 2012;6(2):137–92. 10.1007/s11682-012-9156-5 22438191 PMC3803157

[4] Park E, Ai J, Baker AJ. Cerebellar injury: clinical relevance and potential in traumatic brain injury research. Prog Brain Res 2007;161:327–38. 17618988 10.1016/S0079-6123(06)61023-6

[5] Potts MB, Adwanikar H, Noble-Haeusslein LJ. Models of traumatic cerebellar injury. Cerebellum 2009;8(3):211–21. 10.1007/s12311-009-0114-8 19495901 PMC2734258

[6] Spanos GK, Wilde EA, Bigler ED, Cleavinger HB, Fearing MA, Levin HS, et al cerebellar atrophy after moderate-to-severe pediatric traumatic brain injury. AJNR Am J Neuroradiol 2007;28(3):537–42. 17353332 PMC7977845

[7] Louis ED, Lynch T, Ford B, Greene P, Bressman SB, Fahn S. Delayed-onset cerebellar syndrome. Arch Neurol 1996;53(5):450–4. 8624221 10.1001/archneur.1996.00550050080027

[8] Iwadate Y, Saeki N, Namba H, Odaki M, Oka N, Yamaura A. Post-traumatic intention tremor—clinical features and CT findings. Neurosurg Rev 1989;12 Suppl 1:500–7. 2812421 10.1007/BF01790695

[9] Eierud C, Craddock RC, Fletcher S, Aulakh M, King-Casas B, Kuehl D, et al Neuroimaging after mild traumatic brain injury: Review and meta-analysis. Neuroimage Clin 2014;4:283–94. 10.1016/j.nicl.2013.12.009 25061565 PMC4107372

[10] Mac Donald CL, Johnson AM, Cooper D, Nelson EC, Werner NJ, Shimony JS, et al Detection of blast-related traumatic brain injury in U.S. military personnel. N Engl J Med 2011;364(22):2091–100. 10.1056/NEJMoa1008069 21631321 PMC3146351

[11] Alhilali LM, Yaeger K, Collins M, Fakhran S. Detection of central white matter injury underlying vestibulopathy after mild traumatic brain injury. Radiology 2014;272(1):224–32. 10.1148/radiol.14132670 24735411

[12] Wang JY, Bakhadirov K, Abdi H, Devous MD Sr., Marquez de la Plata CD, Moore C, et al Longitudinal changes of structural connectivity in traumatic axonal injury. Neurology 2011;77(9):818–26. 10.1212/WNL.0b013e31822c61d7 21813787 PMC3162636

[13] Mac Donald CL, Dikranian K, Bayly P, Holtzman D, Brody D. Diffusion tensor imaging reliably detects experimental traumatic axonal injury and indicates approximate time of injury. J Neurosci 2007;27(44):11869–76. 17978027 10.1523/JNEUROSCI.3647-07.2007 PMC2562788

[14] Niogi SN, Mukherjee P. Diffusion tensor imaging of mild traumatic brain injury. J Head Trauma Rehabil 2010;25(4):241–55. 10.1097/HTR.0b013e3181e52c2a 20611043

[15] Smith SM, Jenkinson M, Johansen-Berg H, Rueckert D, Nichols TE, Mackay CE, et al Tract-based spatial statistics: voxelwise analysis of multi-subject diffusion data. Neuroimage 2006;31(4):1487–505. 16624579 10.1016/j.neuroimage.2006.02.024

[16] Chen R, Herskovits EH. Graphical-model based morphometric analysis. IEEE Transaction on Medical Imaging 2005;24(10):1237–1248. 10.1109/TMI.2005.854305 16229411

[17] Chen R, Herskovits EH. Graphical-model-based multivariate analysis of functional magnetic resonance data. NeuroImage 2007;35:635–647. 17258473 10.1016/j.neuroimage.2006.11.040 PMC2427148

[18] Ellison-Wright I, Nathan PJ, Bullmore ET, Zaman R, Dudas RB, Agius M, et al Distribution of tract deficits in schizophrenia. BMC Psychiatry 2014;14(1):99. 24693962 10.1186/1471-244X-14-99 PMC4108049

[19] ACRM. Definition of mild traumatic brain injury. Journal of Head Trauma Rehabilitation 1993;8(3):86–87.

[20] Haacke EM, Duhaime AC, Gean AD, Riedy G, Wintermark M, Mukherjee P, et al Common data elements in radiologic imaging of traumatic brain injury. J Magn Reson Imaging 2010;32(3):516–43. 10.1002/jmri.22259 20815050

[21] Gershon RC, Cella D, Fox NA, Havlik RJ, Hendrie HC, Wagster MV. Assessment of neurological and behavioural function: the NIH Toolbox. Lancet Neurol 2010;9(2):138–9. 10.1016/S1474-4422(09)70335-7 20129161

[22] Jenkinson M, Beckmann CF, Behrens TE, Woolrich MW, Smith SM. FSL. NeuroImage 2012;62:782–90. 10.1016/j.neuroimage.2011.09.015 21979382

[23] Chen R, Herskovits EH. Graphical model based multivariate analysis (GAMMA): an open-source, cross-platform neuroimaging data analysis software package. Neuroinformatics 2012;10(2):119–27. 10.1007/s12021-011-9129-7 21882083 PMC6201747

[24] Mendoza J, Foundas A. Clinical Neuroanatomy: A Neurobehavioral Approach. New York City: Springer; 2007.

[25] Mori S, Oishi K, Jiang H, Jiang L, Li X, Akhter K, et al Stereotaxic white matter atlas based on diffusion tensor imaging in an ICBM template. Neuroimage 2008;40(2):570–82. 10.1016/j.neuroimage.2007.12.035 18255316 PMC2478641

[26] Gale SD, Baxter L, Roundy N, Johnson SC. Traumatic brain injury and grey matter concentration: a preliminary voxel based morphometry study. J Neurol Neurosurg Psychiatry 2005;76(7):984–8. 15965207 10.1136/jnnp.2004.036210 PMC1739692

[27] Lupi A, Bertagnoni G, Salgarello M, Orsolon P, Malfatti V, Zanco P. Cerebellar vermis relative hypermetabolism: an almost constant PET finding in an injured brain. Clin Nucl Med 2007;32(6):445–51. 17515750 10.1097/RLU.0b013e3180537621

[28] Liu W, Wang B, Wolfowitz R, Yeh PH, Nathan DE, Graner J, et al Perfusion deficits in patients with mild traumatic brain injury characterized by dynamic susceptibility contrast MRI. NMR Biomed 2013;26(6):651–63. 10.1002/nbm.2910 23456696

[29] Schmahmann JD, Sherman JC. The cerebellar cognitive affective syndrome. Brain 1998;121 (Pt 4):561–79. 9577385 10.1093/brain/121.4.561

[30] Schmahmann JD, Caplan D. Cognition, emotion and the cerebellum. Brain 2006;129(Pt 2):290–2. 16434422 10.1093/brain/awh729

[31] Ravizza SM, McCormick CA, Schlerf JE, Justus T, Ivry RB, Fiez JA. Cerebellar damage produces selective deficits in verbal working memory. Brain 2006;129(Pt 2):306–20. 16317024 10.1093/brain/awh685

[32] Chu Z, Wilde EA, Hunter JV, McCauley SR, Bigler ED, Troyanskaya M, et al Voxel-based analysis of diffusion tensor imaging in mild traumatic brain injury in adolescents. AJNR Am J Neuroradiol 2010;31(2):340–6. 10.3174/ajnr.A1806 19959772 PMC7964133

[33] Mayer AR, Ling J, Mannell MV, Gasparovic C, Phillips JP, Doezema D, et al A prospective diffusion tensor imaging study in mild traumatic brain injury. Neurology 2010;74(8):643–50. 10.1212/WNL.0b013e3181d0ccdd 20089939 PMC2830922

[34] Henry LC, Tremblay J, Tremblay S, Lee A, Brun C, Lepore N, et al Acute and chronic changes in diffusivity measures after sports concussion. J Neurotrauma 2011;28(10):2049–59. 10.1089/neu.2011.1836 21864134

